# Anomalous chemically induced electron spin polarization in proton-coupled electron transfer reactions: insight into radical pair dynamics†
†Dedicated to Professor Michael R. Wasielewski on the occasion of his 70^th^ birthday.


**DOI:** 10.1039/d0sc02691c

**Published:** 2020-05-28

**Authors:** Alexander M. Brugh, Malcolm D. E. Forbes

**Affiliations:** a Department of Chemistry , Center for Photochemical Sciences , Bowling Green State University , Bowling Green , OH 43403 , USA . Email: forbesm@bgsu.edu

## Abstract

Time-resolved electron paramagnetic resonance (TREPR) spectroscopy has been used to study the proton coupled electron transfer (PCET) reaction between a ruthenium complex (Ru(bpz)(bpy)_2_) and several substituted hydroquinones (HQ). After excitation at 355 nm, the HQ moiety forms a strong hydrogen bond to the exposed N atoms in the bpz heterocycle. At some point afterwards, a PCET reaction takes place in which an electron from the O atom of the hydrogen bond transfers to the metal center, and the proton forming the hydrogen bond remains on the bpz ligand N atom. The result is a semiquinone radical (HQ˙), whose TREPR spectrum is strongly polarized by the triplet mechanism (TM) of chemically induced dynamic electron spin polarization (CIDEP). Closer examination of the CIDEP pattern reveals, in some cases, a small amount of radical pair mechanism (RPM) polarization. We hypothesize that when the HQ moiety has electron donating groups (EDGs) substituted on the ring, S–T^–^ RPM polarization is observed in HQ˙. These anomalous intensities are accounted for by spectral simulation using polarization from S–T^–^ mixing. The generation of S–T^–^ RPM is attributed to slow radical separation after PCET due to stabilization of the positive charge on the ring by EDGs. Results from a temperature dependence support the hypothesis.

## Abstract 2

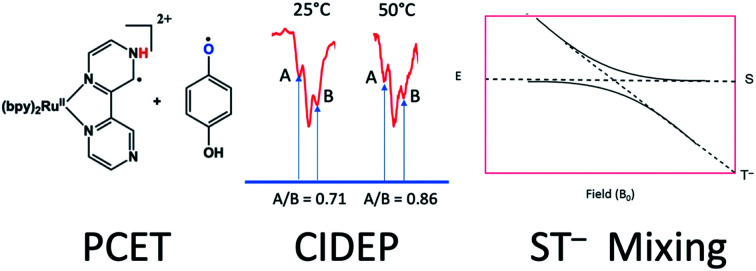
Time-resolved electron paramagnetic resonance (TREPR) spectroscopy has been used to study the proton coupled electron transfer (PCET) reaction between a Ruthenium complex (Ru(bpz)(bpy)_2_) and several substituted hydroquinones (HQ).

## Introduction

Proton-coupled electron transfer (PCET) reactions are important to many naturally occurring chemical processes.^1^ They are also observed as part of overall mechanisms of non-natural chemical reactions, *e.g.* thermal and photoinduced catalysis reactions and redox processes such as in artificial photosynthesis^2^ and fuel cells.^3^ During past two decades, there has been considerable effort to engineer chemical systems that can mimic plant photosystem II (PSII) for possible alternative energy applications^4^ as well as solar fuels technology.^5^ The overall PSII mechanism involves a cascade of PCET reactions that shuttle four electrons and four protons to the correct location in the chloroplast for water oxidation.^6^ The PCET cascade help avoid high-energy reactive intermediates by building multiple redox equivalents at a single site. Therefore, mechanistic details and structure-reactivity studies of the reactive intermediate involved in thermal and excited state PCET reactions remain a research topic of high interest.

**Scheme 1:**
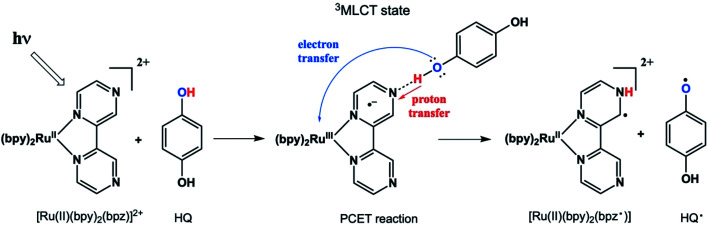


We recently reported a detailed study of a photoinduced PCET reaction involving [Ru(ii)(bpy)_2_(bpz)]^2+^ (bpy = *N*,*N*′-bipyridine, bpz = *N*,*N*′-bipyrazine) with hydroquinone (HQ) (Scheme 1).^7^,^8^ This reaction starts with the photoexcitation of Ru(ii)(bpy)_2_(bpz) into a metal to ligand charge transfer (MLCT) state involving exclusively the bpz ligand. The creation of the ^3^MLCT* excited state dramatically increases the electron density on the ligand,^9^ rendering the non-coordinated nitrogen on the ligand more basic. This higher basicity leads to a hydrogen bonded complex from one of the HQ oxygen atoms to one of the bpz ligand nitrogens. There is no interaction between these two species in the ground state. The H-bonded complex lives for some time before a PCET event leads to the formation of two free radicals. During this process, the H-bonding HQ proton is transferred to the bpz ligand nitrogen, and an electron is transferred from the HQ oxygen to the Ru(iii) metal center. The PCET event destroys the H-bond, and the complex breaks apart. The overall result is a Ru(ii) compound with a radical center formally on the bpz ligand nitrogen, as well as a semiquinone radical HQ˙.

In our two previous papers, this reaction was studied by ultrafast transient absorption and time-resolved electron paramagnetic resonance (TREPR) spectroscopy.^7^,^8^ Solvent, pH, and substituent effects on both the ligand and the HQ were investigated. Also, very strong chemically induced dynamic electron polarization (CIDEP)^10^ was observed in all spectra. The intense CIDEP and relatively slow electron spin relaxation times of HQ˙ allowed us to observe TREPR signals as long as 2 μs after the laser flash. A consequence of these long observation times is that very spectral high resolution was obtained at delay times greater than 1 μs (more details below), enabling the observation of hyperfine couplings of less than 1 Gauss in many cases.

We observed several TREPR spectra with anomalous CIDEP intensities in some of the smaller hyperfine mulitplets. At the time, it was speculated that this anomaly might be connected to the rate at which the radicals separate into free solution after the PCET event. In this paper, we reexamine these anomalous intensities and put forward a hypothesis that indeed connects them to the rate of radical separation through the phenomenon of radical pair mechanism spin polarization induced by S–T^–^ mixing.^11^ Reports of S–T^–^ RPM polarization are rare, and quite special circumstances are required for its observation, *e.g.*, excited state lifetime, hyperfine coupling constants, complex lifetime, and translational diffusion rates all must fall together in certain windows. Below, we present results that provide some insight into the role of PCET in the radical separation process.

## Materials and methods

Ru(iii)bpz(bpy)_2_PF_6_ was synthesized as described previously.^12^ HQ and HPLC grade acetonitrile were purchased from Sigma-Aldrich. The HQ was recrystallized twice from ethanol and dried in a dessicator before use. Acetonitrile was dried over calcium hydride before use. Our X-band TREPR apparatus is described in detail elsewhere.^13^ Briefly, samples were flowed through a 0.4 mm path length Suprasil quartz flat cell with dry nitrogen gas bubbling through a 25 mL reservoir. Flow was necessary to prevent heating of the sample by the source of excitation, a YAG laser (9 ns pulse width, 10 mJ pulse energy at the sample, 30 Hz repetition rate). A boxcar signal averager with 100 ns gates was used to trap the light and dark EPR signals directly from the preamplifier of the microwave bridge. A tuned TE_110_ rectangular microwave resonator with an optical transmission feature was used to enable irradiation of the sample. The microwave power was typically 10 mW and the scan times were 2–4 minutes.

## Results and discussion

**Fig. 1:**
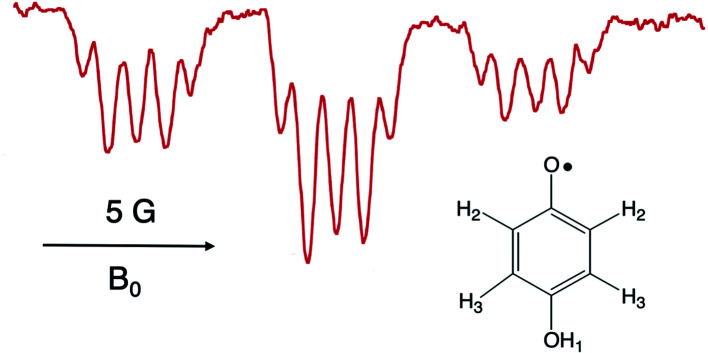
X-band TREPR spectrum acquired 1 μs after a 10 ns, 355 nm laser pulse firing on the PCET system shown in Scheme 1 (150 μL Ru(iii)bpz(bpy)_2_PF_6_, 0.1 M HQ). The spectrum was collected in dry acetonitrile at room temperature, and is assigned to the HQ˙ structure shown. Hyperfine coupling constants are as follows: H_2_ (*ortho*, 5.66 Gauss, triplet), H_3_ (*meta*, 0.72 Gauss, triplet), OH_1_ (1.62 Gauss, doublet).

It is worthwhile to describe a representative TREPR spectrum from the system in Scheme 1 in detail, in order to understand how the different CIDEP mechanisms manifest themselves. Fig. 1 shows X-band TREPR data acquired 1 μs after a 355 nm laser flash of the system shown in Scheme 1. The data were acquired in dry acetonitrile (ACN) as the solvent, to move the equilibrium between HQ˙ and its conjugate base, the quinone radical anion (Q^–^˙). This ensures that only HQ˙ is present at the time of observation. In aqueous solution or wet ACN, both HQ˙ and Q^–^˙ are detected and their spectra overlap, complicating assignments. The spectrum in Fig. 1 is easily simulated as a triplet of triplets of doublets, as all hyperfine splittings are resolved (values are given in the figure caption), including the smallest coupling constant of 0.72 G.^14^ Such high spectral resolution is uncommon in TREPR spectroscopy, because of the 1/*f* noise filtering feature of the boxcar. Basically, the closer one moves the light sampling gate to the laser flash, the less spectral resolution is obtained due to uncertainty broadening (as well as T_2_ if the light sampling gate is very close in time to the flash). It is also important to note that in order to improve the time resolution, TREPR experiments in direct detection mode do not use 100 kHz external field modulation. Therefore, TREPR spectral line shapes are close to Lorentzian rather than the “normal” first derivative shape from steady-state EPR.

**Scheme 2:**
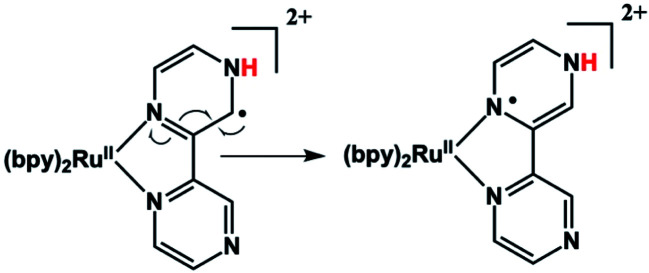


An immediate issue that arises from Fig. 1 is the absence of any TREPR signal from the counter radical, *i.e.*, Ru(iii)(bpy)_2_bpz˙. This species appears to be EPR silent and has not been observed at any delay time in any solvent, even at high concentrations. It is highly likely that this is due to fast electron spin relaxation in this species, because Ru has a reasonably high spin–orbit coupling coefficient.^15^ The unpaired electron in Ru(iii)(bpy)_2_bpz˙ is highly delocalized, and there are several resonance contributors that place the radical center adjacent to the metal center, one example of which is illustrated in Scheme 2. With contributions from such resonance contributors, one would expect significant π and σ through-bond coupling, and therefore very efficient electron spin relaxation in Ru(iii)(bpy)_2_bpz˙.

**Fig. 2:**
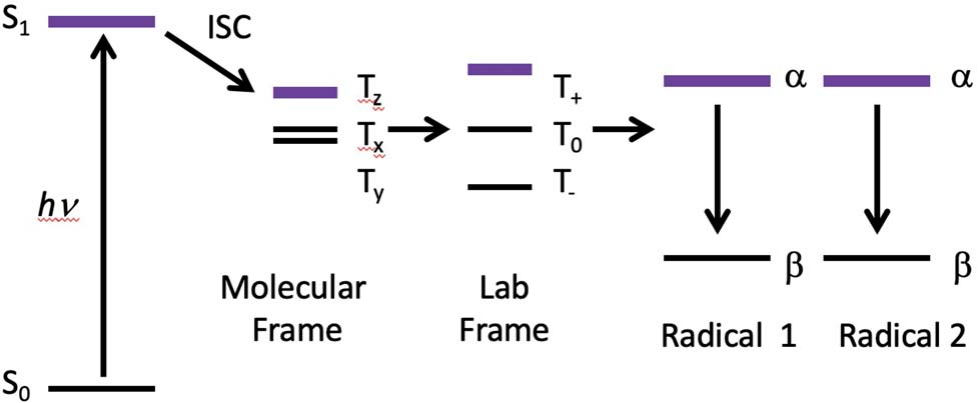
The Triplet Mechanism. A molecule undergoes photoexcitation and intersystem crossing to a triplet state. Due to the anisotropic shape of the molecule, zero-field states are populated selectively at different rates. This polarization is transferred to the laboratory frame and then to any doublet state free radical species created by the photochemistry.

The possibility of fast spin relaxation raises an additional question: if relaxation is so efficient, why is there any TREPR signal observed at all? The answer to this lies in the very intense spin polarization (CIDEP) observed, to which we now turn our attention. The TREPR spectrum in Fig. 1 shows a strong net emissive signal due to the triplet mechanism (TM) of CIDEP. The origin of this polarization is illustrated in Fig. 2. As the name implies, a photoexcited triplet state is involved. The photophysics of Ru complexes has been extensively studied,^16^ and the initial excitation from S_0_ to S_1_ is first of all very rapid, but also results in the immediate conversion to triplet state *via* intersystem crossing.^17^ Rapid ISC is also attributed to the large spin–orbit coupling coefficient associated with the Ru metal center.

The ISC process is anisotropic in the molecular frame, which means that the zero field triplet sublevels (T_*x*_, T_*y*_, and T_*z*_) in this frame are populated selectively at different rates. Moving to the laboratory frame (T^+^, T^0^, and T^–^), the TM polarization is retained. The two frames are simply a rotation or change of basis sets, and so some coefficient of the most heavily populated zero-field sublevel will be transferred to a large (but perhaps not equally large) coefficient of one of the high field sublevels. If the ensuing doublet state free radicals are formed quickly, *i.e.*, before complete electron spin relaxation in the triplet, this polarization will then appear in the TREPR spectrum.

Molecular excited triplet states are not typically observable by EPR spectroscopy in solution at room temperature due to fast spin relaxation. But, we can investigate triplets in solution by examining the spin polarization of the radicals they produce. In general, large anisotropic triplets show very strong TM, sometimes up to 80–100% spin polarized. Since the Boltzmann ratio for equilibrium EPR spin states is 0.17%, it takes a very long time for a large polarization to relax completely. While we may only be observing 2–3% of the original polarization, this is still more than 10 times the Boltzmann value and will be easily detected by TREPR. This explains the strong TM polarization observed here: the anisotropic ^3^MLCT* state generates a large polarization, the non-equilibrium spin state populations relax rather slowly due the large size of the compound, *i.e.*, its tumbling rate in solution is slow.

The concept of “slow” relaxation in the ^3^MLCT* state and “fast” relaxation in the ensuing doublet state of the Ru(iii)(bpy)_2_bpz˙ radical are not contradictory. The time scales for these two processes differ by nearly three orders of magnitude. Electron spin relaxation in the triplet manifold in solution is typically 1–10 ns for a small organic molecule, but could be extended to 50–100 ns for a larger, slower tumbling species. The relaxation mechanism in this case is due to fluctuations in the electron dipole–dipole interaction in the triplet, which depends on the alignment of the dipoles with the applied external magnetic field.^18^ The spin relaxation mechanism for the Ru(iii)(bpy)_2_bpz˙ species is driven by spin–orbit coupling with the Ru metal center, and will be largely independent of the tumbling rate. The electron spin relaxation time for a typical doublet state organic radical in free solution is 1–10 μs, but with large spin–orbit coupling coefficients this can become as fast as 100 ps to 1 ns.

Another nuance of the CIDEP pattern display in Fig. 1 is that the net emissive (E) polarization is not the same for the low field packet of lines *vs.* the high field packet. The low field lines exhibit slightly more E intensity that the high field lines. We attribute this to a small amount of S–T^0^ radical pair mechanism (RPM) CIDEP, which arises from reencounters or doublet state radicals after some hyperfine-induced spin wave function evolution when they are long distances apart.^19^ A careful distinction between S–T^0^ RPM and S–T^–^ RPM will be put forward below; for the present discussion we note that the predicted S–T^0^ RPM pattern would be E/A, that is, low field E, high field A, exactly as observed. The dominance of the TM has been explained above. The weak RPM observed here is most likely due to the slow translational diffusion (fewer reencounters) of the larger Ru(iii)(bpy)_2_bpz˙ radical.

**Fig. 3:**
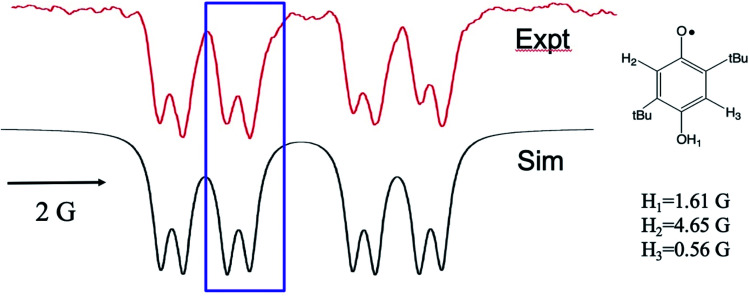
X-band TREPR spectrum (top) and spectral simulation using the TM and hyperfine coupling constants shown (bottom) for the semiquinone radical shown. Experimental conditions are identical to those in Fig. 1 except that the solvent was ACN/water 50 : 50. This HQ˙ radical does not deprotonate in water due to the destabilization of the conjugate base (quinone radical anion) by the alkyl groups, hence the OH_1_ hyperfine is observed for this system.

We now turn to the anomalous intensities observed for certain substituted HQs in this chemistry, namely those with electron donating groups on the ring. An example is presented in Fig. 3, where ^*t*^butyl substituents are present. A spectral simulation is included below the experimental spectrum, which, for the purpose of discussion, involves only the TM. In this figure we have highlighted the discrepancy with boxes: each multiplet in the spectrum shows a more intense high-field line than its low field partner. The phenomenon is observed across the spectrum and only for the smallest splitting. This is a very unusual pattern and, to best of our knowledge, it has not been observed in high resolution TREPR spectra before. Furthermore, we are unable to simulate the anomaly using the pure TM, pure S–T^0^ RPM, or any superposition of the two mechanisms. Clearly there is another mechanism at work here.

**Fig. 4:**
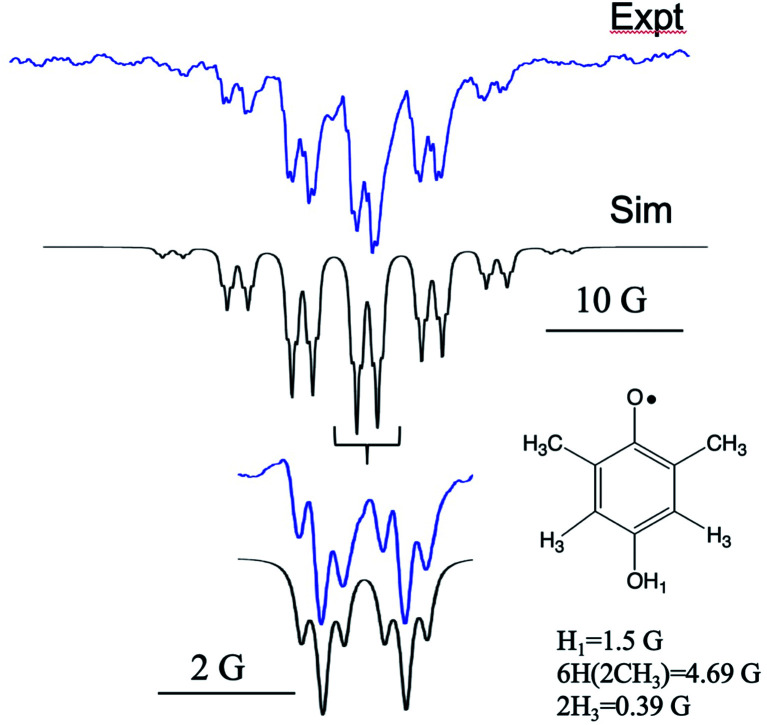
X-band TREPR spectrum (top) and spectral simulation using the TM and hyperfine coupling constants (bottom) for the semiquinone radical shown. Experimental conditions are identical to those in Fig. 1 except that the solvent was ACN/water 50 : 50. This HQ˙ radical does not deprotonate in water due to the destabilization of the conjugate base (quinone radical anion) by the alkyl groups, hence the OH_1_ hyperfine is observed for this system.

Fig. 4 shows another example, this time with methyl substituents on the HQ ring. The experimental spectrum (top) in this case has many more lines in it due to the additional number of protons engaging in hyperfine coupling and some symmetry issues. The overall spectrum consists of a heptet from the 6 equivalent methyl groups, each member of which is split into a doublet by the OH_1_ proton. These in turn experience a small splitting into an additional triplet from the *meta* protons H_3_. These *meta* proton splittings of 0.39 Gauss are among the smallest ever resolved for a TREPR spectrum. Each of the small *meta* triplets also exhibits the same anomalous intensities at seen in Fig. 3, *i.e.*, the low field line of each small triplet is less intense than its high field counterpart. The simulation (center) again cannot account for this pattern using pure TM, pure S–T^0^ RPM, or any combination of the two. The center two triplets in the spectrum are expanded at the bottom of Fig. 4 to best illustrate the anomaly.

**Fig. 5:**
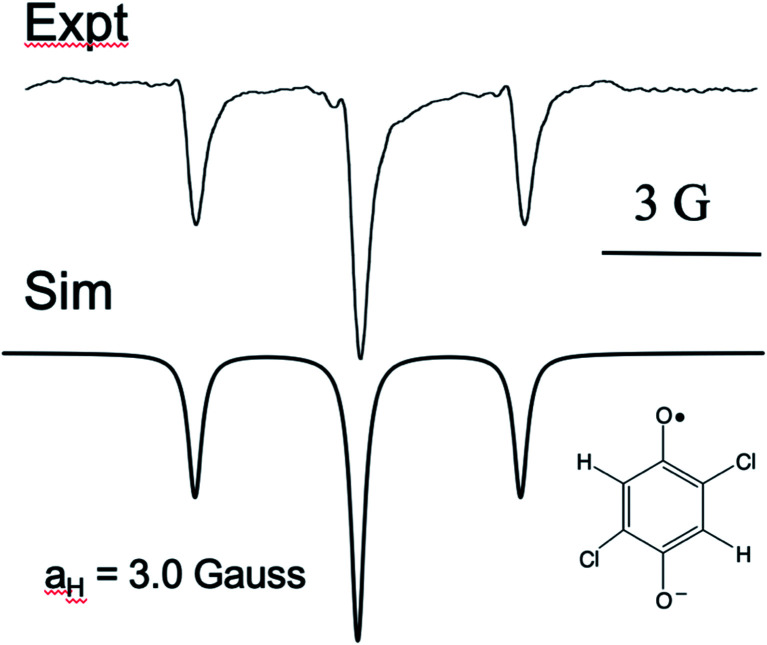
X-band TREPR spectrum (top: experimental) of the quinone anion radical shown. Bottom: Simulation using the hyperfine coupling constant shown and only TM spin polarization. Note that the intensities of all transitions are a perfect match.

It is curious that both observations of these unusual polarization patterns took place with electron donating groups on the ring, and this provided a clue as to the mechanism. To amplify this point, Fig. 5 shows a TREPR spectrum for a similar system except that electron withdrawing groups, namely Cl, are on the HQ ring. The spectrum is symmetric and simple, with two equivalent protons showing hyperfine interaction. The 1 : 2 : 1 triplet observed has a coupling constant of 3 Gauss, and we note that in this case the species observed is the quinone radical anion, because HQ˙ here rapidly deprotonates due to stabilization of the negative charge on the conjugate base. Most notable here is that the 1 : 2 : 1 triplet is precisely symmetric in intensities of the low field and high field lines. There is not even any S–T^0^ RPM polarization, just pure TM. We note also that the multiplets in the parent compound, HQ with only hydrogen atoms on the ring, is also symmetric (Fig. 1). It is clear that electron donating groups are necessary to observe the unusual intensities.

**Fig. 6:**
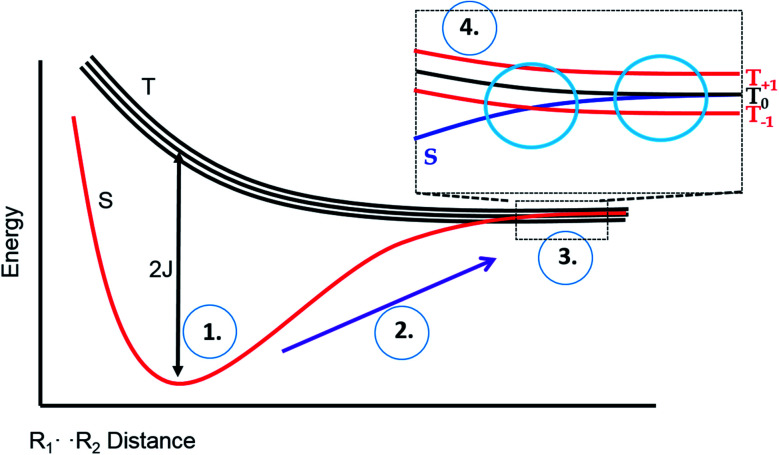
Energy level diagram for the S and T spin states of a radical pair in solution, as a function of inter-radical distance. It is implied that an external magnetic field is present, slightly separating the energies of the three high field triplet levels discussed in Fig. 2. (1) The initial position of a photogenerated radical pair, with a large gap between S and T. (2) The pair diffuse apart for where the S–T gap (2*J*) is small, (3) mixing of the S and T state can occur, after which the radical pair may undergo a reencounter. (4) Insert indicates a level crossing of the S and T– levels (left blue circle), and the region of S–T^0^ mixing (right blue circle).

**Fig. 7:**
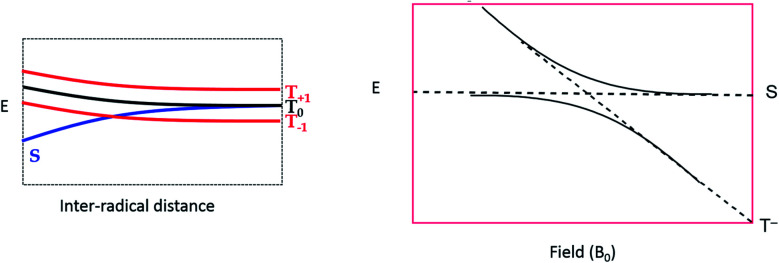
The crossing region for S–T-mixing. Left: S and T levels in an applied magnetic field, non-interacting. Right: expansion of the crossing region showing that some radical pairs will cross without interacting, but others will undergo an avoided crossing, and switch manifolds from T to S or *vice versa*.

To explain these observations, it is instructive to highlight the difference between the S–T^0^ RPM and the S–T^–^ RPM polarization mechanisms. Fig. 6 provides some insight. Here we show the energy levels of the radical pair as a function of inter–radical distances. It should be noted that the vertical scale of this diagram is very small compared to, say the energies of chemical bonds. Were this plotted on the same scale as, for example, a Jablonski diagram for a normal organic molecule, this entire plot would barely take up one pixel. Radical pairs are not electronically excited. Rather, an excited state has dissipated its energy to create them. The energy gap between the singlet and the triplet is defined as 2*J*, where *J* is the exchange interaction between the unpaired spins. At very close contact *J* is large and fast spin exchange tends to lock the spin state of the radical pair (S or T). But at long distances such as on the far–right blue circle, *J* is small and the S and T^0^ states in particular begin to mix *via* local magnetic field differences (nuclear hyperfine interactions and *g* factor differences). When some of this spin wave function evolution has occurred, if a reencounter or the radicals takes place *via* diffusion, the radical pair must choose to be in the S manifold or the T^0^ manifold. This polarizes the spins, either E/A or A/E, and is called the S–T^0^ RPM. The difference between S and T^0^ in the mixing region is simply a phase relationship – they are energetically degenerate, which is why they mix to create new eigenstates (Fig. 7).

There is another region in the diagram where S states and T states can mix, shown in the left blue circle of region 4 in Fig. 6. Because *J* is negative in sign, there is a region of radical separation where S and the T^–^ level are subject to an avoided crossing. For some states, mixing is symmetry allowed *via* a second order process. Some energy levels are forbidden to mix due to selection rules and this forms the crux of our argument for the anomalous intensities observed in Fig. 3 and 4. There are two relevant equations. From a chemical reaction kinetics perspective, the rate at which a T^–^ state converts to S can estimated *via* perturbation theory. The rate of reaction of the T^–^ level depends on how much “S” character it picks up in the crossing region. This is given by the following expression:

1 *k*_T^–^_ = *k*_r_*λ*_ST^–^_ = *k*_r_[*a*^2^/(*a*^2^ + *J*^2^)]

Here *k*_r_ is the radical reencounter rate and *λ*_ST^–^_ is the fraction of singlet character acquired during the avoided crossing. The term *λ*_ST^–^_ is obtained from perturbation theory (mixing element squared divided by the square of the energy gap).^20^ Because the transition from T^–^ to S requires a change in the total spin from –1 to 0, there must be a corresponding change in angular momentum elsewhere, for conservation. Therefore, the nuclear spin involved in the mixing must change from a higher value to a lower value, *i.e.*, there is a selection rule. We can therefore estimate the magnitude of RPM S–T^–^ CIDEP, *P*_S–T^–^_, from the following equation:

2 *P*_S–T^–^_ ∼ *a*_H_^2^[*I*(*I* + 1) – *m*(*m* + 1)]

Here *I* represents the total nuclear spin quantum number and *m* is the nuclear sub-level spin quantum number, and *P*_S–T^–^_ is the polarization which is directly proportional to the transition intensity.

A consequence of the selection rule is that there will always be, in any hyperfine multiplet, a so–called “orphan” state, *i.e.*, an electron nuclear spin sub-level that cannot mix from T^–^ to S. If we start from a pure triplet state, the S–T^–^ mixing process populates S and depopulates T^–^. As mentioned above, the net change in total spin for the radical pair in this process is +1. Nuclear spin sublevels can change correspondingly by –1, except for the lowest total spin quantum number. It cannot change beyond its current value without violating the selection rule, and so it cannot be polarized by the S–T^–^ RPM.

**Fig. 8:**
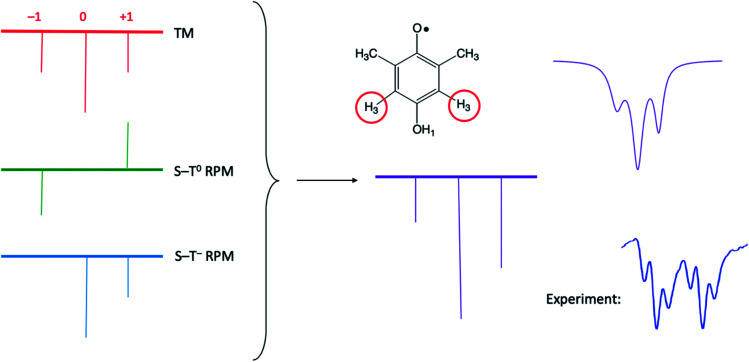
Stick plots of the three different CIDEP mechanisms present in Fig. 4 for dimethyl HQ. Top left: The TM, with symmetric intensities. Top middle: The S–T^0^ RPM, showing E/A polarization. Left bottom: The S–T^–^ RPM, showing zero intensity for the orphan state at low field that is forbidden from mixing by this mechanism. Center: addition of all three stickplots. Top right: summed spectra with added Lorentzian line shape, which compares very favorably to the experimentally observed line shape (bottom right).

It is very helpful to close out our discussion with an example, which is presented in Fig. 8. Here we are simulating only one of the multiplets from the bottom of Fig. 4 (dimethyl HQ). On the left are stick plots showing the expected pattern from the TM (top left), the S–T^0^ RPM (middle left), and the S–T^–^ RPM (bottom left). For TM there is a net emissive spectrum that gives a 1 : 2 : 1 intensity pattern on these peaks. For S–T^0^ RPM there is an approximate –1 : 0 : 1 pattern, low field emissive and high field absorptive. However, for S–T^–^ RPM the resulting intensities are 0 : 2 : 1, due to the spin forbidden transition. In the center of Fig. 8 these on the left, with very little RPM added, as we know there is not much of this mechanism present in the experimental spectrum. Note that a superposition of the top and middle stick plots will never reproduce the experiment, because the high field line will always be less intense than the low field line (the high field lines have opposite polarizations and will cancel each other). See Fig. 8 caption for further details.

**Fig. 9:**
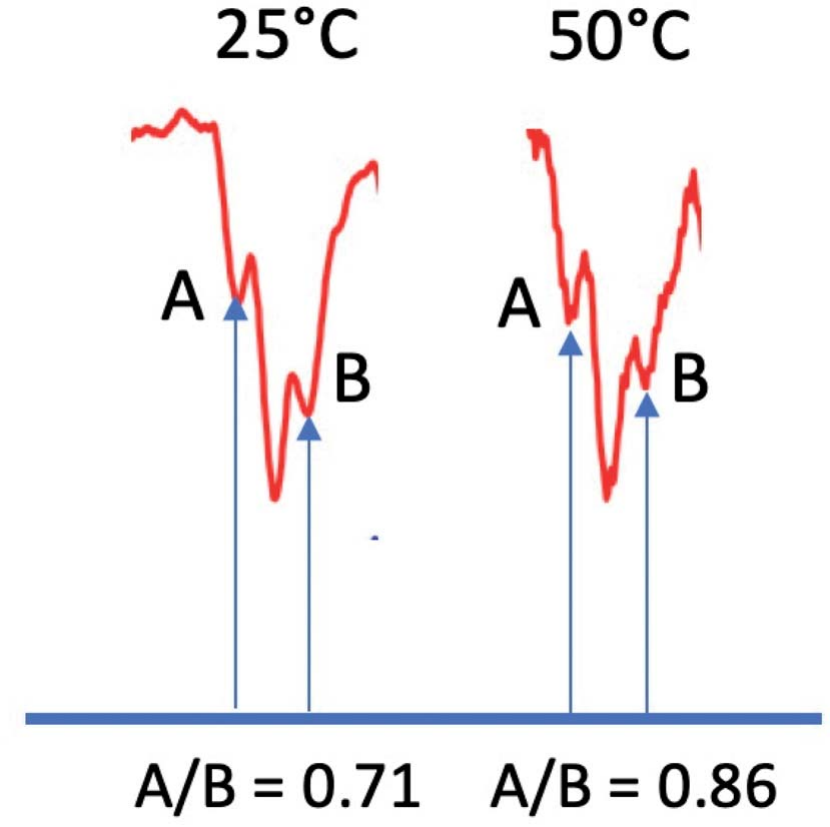
Ratios of outer peak heights as a function of temperature for the central multiplet of the dimethyl HQ semiquinone radical.

Since ST^–^ mixing will be strongly dependent on the time spent in the crossing region, temperature should have an effect on these anomalous intensities. To this end, we compared datasets at 25 °C and 50 °C. The results, obtained at a delay time of 1 μs at the same concentrations and same solvent systems as before, are shown for one multiplet in Fig. 9. Higher temperatures should speed up time spent in the mixing region and reduce the magnitude of the anomaly. Indeed, this is observed. Because the natural line width should not change much between the temperatures selected (and even then, should become narrower at the higher temperature value), we use simple peak height ratios to illustrate this quantitively. The ratio of the peak heights on the low field to high field side (peak A and peak B) has a ratio of 0.71 at 25 °C and moves closer to equal at 50 °C, to 0.86. This is an overall change of about 20%, which is a surprisingly significant effect.

**Scheme 3:**
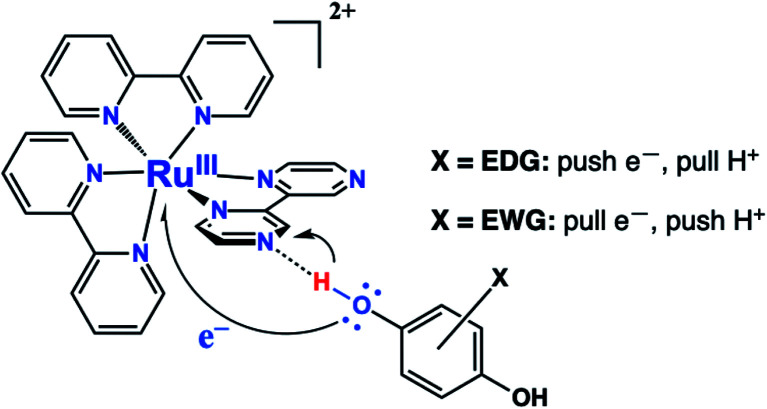


A final point of discussion is the substituent effect, *i.e.*, why do we observe this unusual polarization only with EDGs? What makes significant time in the S–T^–^ mixing region more likely in such cases? Scheme 3 shows the role substituents play in the PCET mechanism. Clearly, EDGs will destabilize negative charges and therefore they will promote the transfer of the electron from the HQ oxygen atom to the metal. Correspondingly, they will stabilize the small positive charge on the H atom, they may also donate electron density to toward the H-bond. So, there will be a tendency for a “pull” on the H and a “push” on the electron.^21^ The opposite will be true for EWGs. Our results suggest that with EDGs, the electron transfer portion of the PCET reaction is moving ahead slightly faster, creating an electron deficient oxygen that then leads to a weakening of the H-bond, but slowly so that radical separation leads to time in the mixing region. The role of EWGs should be opposite, in other words protonation of the nitrogen on the bpz ligand moves ahead of electron transfer, leading to different separation dynamics. The time scales for these processes should be on the order of the inverse of the hyperfine coupling constant in frequency units.

It is also worth mentioning the magnitude of the exchange interaction *J* at such distance, which might be expected to be so large as to preclude any mixing at such distances (a few Ångstroms). However, it is well known that in charged radical systems, *J* can flip sign due to configuration interaction with ionic excited states.^22^ Such interactions are offset by overlap integrals, and so overall the magnitude of *J* might be much smaller than expected for electrically neutral systems. This will also affect the value of singlet character acquired during mixing (eqn (1)). A smaller *J* value will increase this significantly, which has ramifications for both chemical reactivity and polarization intensities.

## Conclusions

We have presented a detailed investigation of a radical reaction involving PCET that shows anomalous CIDEP intensities that we attribute to S–T^–^ mixing during radical separation after the PCET event. The phenomenon is observed when EDGs are present on the ring of the HQ participant, which functions as both the H and e^–^ donor. Temperature studies support the hypothesis, and the unusual CIDEP intensities cannot be explained by any other CIDEP mechanism, or combinations thereof. Future work will include additional variations in temperature, viscosity, and substituents, as well as a molecular dynamics simulation of these reactions to better assess the time scales of the proposed events.

## Conflicts of interest

The authors declare no competing interests.

## References

[cit1] Cukier R. I., Nocera D. G. (1998). Annu. Rev. Phys. Chem..

[cit2] Hammarström L., Hammes-Schiffer S. (2009). Acc. Chem. Res..

[cit3] O'Regan B., Grätzel M. (1991). Nature.

[cit4] Dresselhaus M. S., Thomas I. L. (2001). Nature.

[cit5] Gust D., Moore T. A., Moore A. L. (2009). Acc. Chem. Res..

[cit6] Rappaport F., Boussac A., Force D. A., Peloquin J., Brynda M., Sugiura M., Un S., Britt R. D., Diner B. A. (2009). J. Am. Chem. Soc..

[cit7] Concepcion J. J., Brennaman M. K., Deyton J. R., V Lebedeva N., Forbes M. D. E., Papanikolas J. M., Meyer T. J. (2007). J. Am. Chem. Soc..

[cit8] V Lebedeva N., Schmidt R. D., Concepcion J. J., Brennaman M. K., Stanton I. N., Therien M. J., Meyer T. J., Forbes M. D. E. (2011). J. Phys. Chem. A.

[cit9] Yeh A. T., Shank C. V., McCusker J. K. (2000). Science.

[cit10] Harbron E. J. and Forbes M. D. E., in Encyclopedia of Chemical Physics and Physical Chemistry, IOP Publishing, London, 2001, p. 1389.

[cit11] Yeh A. T., Shank C. V., McCusker J. K. (2000). Science.

[cit12] Boully L., Turck A., Plé N., Darabantu M. (2005). J. Heterocycl. Chem..

[cit13] Forbes M. D. E., Jarocha L. E., Sim S., Tarasov V. F. (2013). Adv. Phys. Org. Chem..

[cit14] Uber W. and Stegmann H. B., in Landolt-Börnstein - Group II Molecules and Radicals, ed. H. Fischer and K.-H. Hellwege, Springer-Verlag Berlin Heidelberg, Verlag Berlin Heidelberg, 1979.

[cit15] (b) Dechun Z., in Organic Light-Emitting Diodes, ed. A. Buckley, Woodhead Publishing, 2013, pp. 114–142.

[cit16] Damrauer N. H., Cerullo G., Yeh A., Boussie T. R., Shank C. V., McCusker J. K. (1997). Science.

[cit17] Turro N. J., Modern Molecular Photochemistry, University Press, Menlo Park, CA, 1978.

[cit18] Slichter C. P., Principles of Magnetic Resonance, Springer, Verlag Berlin Heidelberg, 1990.

[cit19] Tarasov V. F., Jarocha L. E., Avdievich N. I., Forbes M. D. E. (2014). Photochem. Photobiol. Sci..

[cit20] Closs G. L., Forbes M. D. E., Norris J. R. (1987). J. Phys. Chem..

[cit21] Ahn D. S., Park S. W., Lee S., Kim B. (2003). J. Phys. Chem. A.

[cit22] Sekiguchi S., Kobori Y., Akiyama K., Tero-Kubota S. (1998). J. Am. Chem. Soc..

